# On the Influence of the Ingestion of Coffee on the Urea and Chlorides in the Urine

**Published:** 1866-05

**Authors:** Charles E. Squarey

**Affiliations:** Resident Medical Officer to the London Fever Hospital


### ON THE INFLUENCE OF THE INGESTION OF COFFEE ON THE UREA AND CHLORIDES IN THE URINE.

By CHARLES E. SQUAREY, M.R.C.S., Resident Medical Officer to the London Fever Hospital.

(COMMUNICATED BY DR. GARROD.)

Observations were made on three cases whilst Mr. Squarey was residing at University College Hospital as physician's assistant. The coffee was taken three times daily, at first in quarter ounce doses, and gradually increased till in the third case from four to six ounces were taken each day. The temperature in two of the cases was taken night and morning, and was never found to be above or below the limits of health. The urine was collected every morning at eight A.M., and examined the same day for urea and chlorides. Both analyses were made by Liebig's volumetric method.

UREA.

In the first case, the observations were carried on for six weeks. The patient's health was good the whole time, and he never complained of any uneasy sensation after taking the coffee. It was taken three times daily in quarter-ounce doses every alternate week. On comparing corresponding weeks of coffee and non-coffee taking, no appreciable difference is to be found. The greatest is in the first and second weeks. The daily average in the second week, when quarter-ounce doses of coffee were taken three times each day, was 2·198 grammes more than in the first week when no coffee was taken; in the third and fourth weeks the daily average was less by ·424 of a gramme in the fourth week when coffee was taken; in the fifth and sixth weeks the daily average was less by .515 of a gramme in the sixth week when no coffee was taken.

In the second case, the observations, owing to an attack of tonsillitis supervening, and the patient leaving the hospital immediately on recovery, were only continued for one week, so that the influence of the coffee can only be judged of by comparing the amount of urea passed per kilogramme of body weight with the normal amount in health. The patient's age was 17 years; his weight was 115 lbs., or 52 kilogrammes; and he passed on the average ·428 of a gramme of urea per kilogramme of body weight. He was taking half-ounce doses of coffee three times daily.

In the third case, the observations were carried on for ten weeks. On comparing the first and second weeks, it is found that the daily average was greater in the second, when three cups of a strong infusion of coffee were taken three times each day, by ·381 of a gramme. On comparing the third and fourth weeks, a great diminution is found in the daily average of the latter, when quarter-ounce doses of coffee were taken three times daily; it was less by 5·099 grammes. Yet in the fifth week, when the same amount of coffee was taken, and in the seventh week, when half an ounce was taken twice a day, and in the eighth week, when half an ounce was taken three times a day, the daily average was greater than in the sixth, when no coffee was taken; so that, although the daily average was lessened one week when quarter-ounce doses of coffee were taken, yet it did not rise when the coffee was left off, or become again diminished when the same and even larger doses were taken. The daily average in the ninth week, when from one and a-half to six ounces of coffee were taken each day, was less by 4·795 grammes than in the tenth week, when no coffee was taken; but in the tenth week, the patient not feeling well, the diet, which up to this time had been very strict, was varied, his health and appetite improved, and with it there was naturally a daily increase in the excretion of urea.

From these results, Mr. Squarey argues that coffee in the above doses certainly does not increase the excretion of urea, or diminish it to any appreciable extent; for he says that the slight difference that occurred in the daily average of the six consecutive weeks in the first case was by no means beyond the limits of health. That in the second case the amount of urea excreted per kilogromme of body weight was quite normal. That although in the third case there was the large diminution of five grammes in the daily average of the fourth week, when coffee was taken, yet this diminution did not recur when the same and even larger doses were taken; nor did the daily average rise when the coffee was left off, which it should have done had the decrease been entirely due to the influence of the coffee. That, according to Dr. Parkes, the decrease of five grammes in the daily average is not beyond the limits of health; for Dr. Parkes, in his book on the Urine, page 8, says that "the maximum and minimum amounts of urea passed on any one day by an individual are usually about one fifth above and below his mean amount;" so that in the third case, the patient passing on the average between thirty and thirty-five grammes, an increase or diminution of six grammes would be within the normal limits.

CHLORIDES.

In the first case, the daily average of the two weeks when coffee was taken in quarter-ounce doses was as nearly as possible the same as in the two corresponding weeks when no coffee was taken; the amount excreted per kilogramme of body weight being quite normal.

In the second case, half-ounce doses of coffee being taken three times daily, the amount of chlorides excreted per kilogramme of body weight was ·162 of a gramme—a rather large, but certainly not abnormal proportion for a boy aged seventeen, and weighing 52 kilogrammes.

In the third case, the rate of excretion of the chlorides was high throughout the whole course of the observations, both during the coffee taking and the non-coffee taking weeks. The amount excreted per kilogramme of body weight was highest in the seventh week, 272 of a gramme, when half-ounce doses of coffee were taken twice daily; and lowest in the eighth week, ·168 of a gramme, when half-ounce doses were taken three times each day.

In conclusion, Mr. Squarey states that neither of the patients suffered in any way from ill effects from taking the coffee. There was never any giddiness, delirium, or unsteadiness of the hands. The patients invariably slept well.

The pulse, noted several times, was generally found to be increased for half an hour or so after taking the coffee.

The President spoke in terms of commendation of the large and patient observations in the author's paper. There were, however, several points in it on which further information was required. In the first place, it was necessary to know more exactly what kind of coffee was used—whether it was Plantation or Mocha, and whether, if it had been bought ground, it had not been adulterated. Again, the amount of exercise taken during the experiments would affect their results considerably. The effect of muscular activity in increasing the amount of urea was shown by its increase in chorea. Coffee would sometimes constipate, and at other would act as an aperient.

Dr. Webster said that what was often sold as coffee might scarcely be coffee, but chickory. His object, however, in rising, was to remark, with great submission, that he could scarcely understand the paper. This was not due to any fault in the Secretary's reading, but to using of French names for weights which required calculation. He had, he remarked, protested before against this, and still thought that English words ought to be used before an English society. He thought also the subject should be studied on a more extensive scale, and suggested to the author to inquire more widely into the effects of coffee-drinking. To do this, he had only to go across the Channel to France, and then to Spain, where no coffee was drunk, but only chocolate.—*London Lancet.*

